# Quality by Design–Driven Formulation Development of Cannabidiol Orally Disintegrating Tablets

**DOI:** 10.1155/sci5/3553253

**Published:** 2026-08-27

**Authors:** Chaowalit Monton, Poj Kulvanich, Apirada Sucontphunt, Jirapornchai Suksaeree, Laksana Charoenchai, Thanapat Songsak

**Affiliations:** ^1^ Drug and Herbal Product Research and Development Center, College of Pharmacy, Rangsit University, Lak Hok Pathum Thani, 12000, Thailand, rsu.ac.th; ^2^ Department of Pharmacognosy, College of Pharmacy, Rangsit University, Lak Hok Pathum Thani, 12000, Thailand, rsu.ac.th; ^3^ Medicinal Cannabis Research Institute, College of Pharmacy, Rangsit University, Lak Hok Pathum Thani, 12000, Thailand, rsu.ac.th; ^4^ Industrial Pharmacy Program, College of Pharmacy, Rangsit University, Lak Hok Pathum Thani, 12000, Thailand, rsu.ac.th; ^5^ Department of Pharmaceutical Chemistry, College of Pharmacy, Rangsit University, Lak Hok Pathum Thani, 12000, Thailand, rsu.ac.th

**Keywords:** anxiety, Box–Behnken design, cannabinoid, design of experiments, orally disintegrating tablets

## Abstract

The development of cannabidiol (CBD) orally disintegrating tablets (ODTs) is effective in treating anxiety in a patient‐friendly manner. The application of Quality by Design (QbD) enhances the efficiency and robustness of the pharmaceutical development of CBD ODTs. The objective of this work was to develop a formulation for CBD ODTs using a QbD‐driven approach. Quality target product profile, critical quality attributes, and an initial risk assessment were identified and evaluated. Subsequently, a Box–Behnken design was employed to analyze the effects of varied compression force, the quantity of microcrystalline cellulose, and the quantity of croscarmellose sodium to create a design space and control space. Results indicated that the design and control spaces produced tablets with hardness ranging from 4 to 6 kg‐force, a disintegration time (DT) ≤ 30 s, and a friability ≤ 1%. All formulations contained 4% CBD (or 10 mg per tablet). The optimal formulation consisted of 35% microcrystalline cellulose and 1% croscarmellose sodium and was compressed at 1400 pounds per square inch. This formulation exhibited a hardness of approximately 5 kg‐force, a DT of 13–15 s, and a friability of approximately 0.3%. Verification data confirmed the accuracy of the predictions made by computer software. The content uniformity and assay determined using validated high‐performance liquid chromatography ranged between 90% and 100%. CBD was released from the CBD ODT in 1% sodium lauryl sulfate solution, with approximately 76% dissolved within 3 h in the dissolution study. In conclusion, the QbD‐driven approach successfully facilitated the formulation development of CBD ODTs with the desired properties for the treatment of anxiety in a patient‐friendly manner.

## 1. Introduction

The therapeutic landscape is experiencing a notable shift with the increasing recognition of cannabidiol (CBD) as a potential treatment for various health conditions [1–6], particularly anxiety disorders [7]. CBD, a Class II Biopharmaceutical Classification System (BCS) substance [8–10] and a nonpsychoactive cannabinoid derived from the *Cannabis sativa* plant, has demonstrated a supportive role in managing anxiety without eliciting the intoxicating effects characteristic of its psychotropic counterpart, tetrahydrocannabinol (THC) [11]. Accordingly, there remains interest in developing patient‐friendly CBD dosage forms with improved pharmaceutical performance and acceptability.

Recently, CBD has been marketed in various dosage forms, including oral solutions (e.g., Epidiolex), wafer tablets (e.g., Xativa), and oral drops. However, oral solutions and oral drops present limitations related to stability, storage, transportation, and microbial contamination, whereas wafer tablets require energy‐intensive freeze‐drying processes with relatively high production costs [12–14]. In this context, orally disintegrating tablets (ODTs) have garnered significant interest as a promising drug delivery modality. ODTs rapidly disintegrate in the oral cavity and may improve convenience and patient acceptability, particularly in patients with swallowing difficulties [15].

Despite the potential benefits, the formulation of CBD ODTs remains challenging. Achieving optimal tablet characteristics—such as sufficient mechanical strength, rapid disintegration time (DT), and minimal friability—requires meticulous consideration of not only the active ingredient but also the selection and proportioning of excipients [16]. Quality by design (QbD) methodology presents a robust framework for addressing these complexities by promoting a systematic approach to formulation development that correlates process variables with product quality attributes [17, 18]. The significance of applying QbD in this context lies in its ability to minimize trial‐and‐error experimentation, enhance reproducibility, and provide a science‐driven rationale for parameter selection. Furthermore, QbD supports risk assessment, aligns formulation development with regulatory expectations, and facilitates future scale‐up, thereby ensuring that the final product is not only therapeutically effective but also consistent and patient‐centric.

Several studies have reported CBD‐containing oral dosage forms and ODTs, focusing mainly on solubility enhancement, excipient compatibility, or formulation feasibility [13, 19–21]. In parallel, CBD ODT compositions have also been disclosed in patent literature. However, these reports mainly focus on formulation feasibility, excipient selection, or solubility enhancement and do not provide a comprehensive pharmaceutical development strategy based on a structured QbD framework. Although previous studies have applied factorial or Design of Experiments (DoE)–based approaches for CBD ODT development [20], limited attention has been given to the integration of Quality Target Product Profile (QTPP) definition, Critical Quality Attributes (CQAs) identification, initial risk assessment, and establishment of both design space and control space within a systematic QbD framework. Moreover, systematic evaluation of formulation and process variables influencing the quality attributes and robustness of CBD ODTs remains limited. Consequently, a regulatory‐aligned and scientifically rigorous development framework for CBD ODTs is still limited in the current literature. The present study, therefore, adopts a structured QbD‐driven approach, integrating risk assessment and DoE to establish both design space and control space for CBD ODT formulation. Rather than focusing solely on formulation feasibility, this work aims to enhance formulation robustness, predictability, and potential scalability. The findings provide not only an optimized CBD ODT formulation but also a comprehensive development strategy that may be extended to future cannabinoid‐based pharmaceutical products.

## 2. Materials and Methods

### 2.1. Materials

CBD standard (purity 99.38%) was obtained from the Department of Medical Sciences, Ministry of Public Health, Thailand. CBD isolate (purity 99.80%) was purchased from RBJ Co., Ltd., Bangkok, Thailand. Spray‐dried mannitol (Mannogem EZ, SPI Pharma, DE, USA) was obtained as a sample from DKSH (Thailand) Ltd., Bangkok, Thailand. Microcrystalline cellulose (MCC, Comprecel M102) was purchased from Maxway Co., Ltd., Bangkok, Thailand. Croscarmellose sodium (CCS) was obtained as a sample from Onimax, Bangkok, Thailand. Fumed silica was purchased from P.C. Drug Center, Bangkok, Thailand. Sucralose and magnesium stearate (MgSt) were purchased from Krungthepchemi Co., Ltd., Bangkok, Thailand. Sodium lauryl sulfate (SLS) was purchased from Kemaus, Elago Enterprises Pty Ltd, New South Wales, Australia. Methanol (HPLC grade) was purchased from Fisher Chemicals (Leicestershire, UK). Water was produced using a Merck Direct‐Q 3UV water purification system (Darmstadt, Germany).

### 2.2. QbD Approach

The QbD‐driven formulation development process for CBD ODTs consisted of QTPP definition, CQAs identification, initial risk assessment, formulation optimization, and evaluation of the optimized formulation. This systematic approach was applied to establish a scientific understanding of the relationships among formulation variables, process parameters, and critical tablet properties. The overall workflow aimed to support the development of CBD ODTs with appropriate mechanical strength, rapid disintegration, and acceptable pharmaceutical performance.

#### 2.2.1. QTPP, CQAs, and Initial Risk Assessment

The QTPP of CBD ODTs was identified across multiple elements. The target and justification for each QTPP element were also provided. Then, the quality attributes of CBD ODTs were identified as CQAs based on their potential impact on safety and efficacy. The target and corresponding justifications for each CQA were also provided. An initial risk assessment for CBD ODTs was conducted, focusing on critical attributes of CBD, and formulation and process parameters. Justifications for each parameter were also provided.

#### 2.2.2. Formulation Design and DoE

A prototype formulation of CBD ODTs, including the ingredients, their quantities, and respective functions for further development, is presented in Table 1. All ingredients were sieved before use: Most components passed through a 60‐mesh sieve, while MgSt and CCS were passed through an 80‐mesh sieve. The formulation was prepared at a laboratory scale sufficient to yield 35 tablets. Initially, CBD, MCC, CCS, fumed silica, and sucralose were blended using the geometric dilution method, with each mixing step conducted for 3 min. Separately, MgSt was premixed with spray‐dried mannitol to enhance the uniform distribution of MgSt within the blend. This premix was then incorporated into the initial mixture and blended for an additional 3 min. The final powder blend was weighed in 250‐mg portions and compressed using an in‐house assembled hydraulic press equipped with a pressure gauge. The die used in this study had an internal diameter of 9.7 mm.

**TABLE 1:** Prototype formulation of CBD ODTs for further development.

Ingredients	Amount per tablet (mg)	Percentage (%)	Function
CBD	10	4	Active pharmaceutical ingredient
Spray‐dried mannitol	127	50.8	Filler
MCC	100	40	Filler/binder
CCS	7.5	3	Superdisintegrant
Fumed silica	2.5	1	Glidant
Sucralose	0.5	0.2	Sweetening agent
MgSt	2.5	1	Lubricant
Total	250	100	

Formulation and process parameters identified as high risk were further investigated using a DoE approach. Compression force, the quantity of MCC, and the quantity of CCS were selected as independent variables and varied according to a Box–Behnken design. Each factor was studied at three levels—low, medium, and high—coded as −1, 0, and +1, respectively, as presented in Table 2. The selected levels were based on findings from a preliminary study. The formulation goals for the development of CBD ODTs are also summarized in Table 2.

**TABLE 2:** Factors and levels, and response and goal used in the Box–Behnken design for optimizing CBD ODTs.

Factors	Levels		
	−1	0	+1
Compression force (psi)	1250	1500	1750
Quantity of MCC (%)	30	40	50
Quantity of CCS (%)	1	3	5

**Responses**	**Goals**		

Individual weight	250 mg		
Thickness	≈ 3 mm		
Diameter	9.7 mm		
Hardness	4−6 kgf		
DT	≤ 30 s		
Friability	≤ 1%		

The tablets prepared according to the Box–Behnken design were evaluated for individual weight, thickness, diameter, hardness, DT, and friability. Tablet hardness was determined using a hardness tester and reported in kilogram‐force (kgf). DT was evaluated by placing each tablet in water maintained at 37 ± 0.5°C and recording the time required for complete disintegration. Friability testing was performed using a friabilator operated at 25 rpm for 4 min, and the percentage weight loss was subsequently calculated. Each test was performed on 10 tablets, except for DT and friability, which were assessed using 6 and 13 tablets, respectively. All testing procedures were conducted in accordance with a previously published study [22].

Tablet properties—specifically hardness, DT, and friability—were analyzed using licensed Design‐Expert software Version 11 (Stat‐Ease Inc., MN, USA) to generate response surface plots, equations (in both coded and actual values), and analysis of variance (ANOVA) data. The design spaces were established based on the following criteria: tablet hardness of 4–6 kgf, DT not exceeding 30 s, and friability not exceeding 1.0%. A control space was defined within the selected design space, and a formulation within this space was designated as the optimal formulation. Three independent lots of the optimal formulation were prepared to verify the accuracy of the model predictions generated by Design‐Expert. The predictions were considered accurate if the actual experimental values closely matched the predicted values and fell within the 95% confidence interval (CI).

#### 2.2.3. Determination of Content Uniformity and Assay

Content uniformity was assessed by individually analyzing the CBD content in each ODT (*n* = 10). Each tablet was placed in a 50‐mL volumetric flask, to which 2 mL of water was added to facilitate tablet disintegration. Approximately 30 mL of methanol was then added, and the mixture was ultrasonicated for 10 min. After the ultrasonication process, the volumetric flask was allowed to cool to room temperature. The volume was adjusted to 50 mL with additional methanol, thoroughly mixed, and then 2.5 mL of this solution was transferred to a 10‐mL volumetric flask. The volume was again adjusted with methanol, mixed thoroughly, and filtered through a 0.45‐μm nylon syringe filter prior to HPLC analysis. The average content (*X̅*), standard deviation (SD), and acceptance value (AV) calculated according to the United States Pharmacopeia (USP) were reported.

For the assay determination, 10 tablets were ground into a fine powder using a mortar and pestle. An accurate weight of 250 mg of the powdered tablets was measured and placed into a 50‐mL volumetric flask (*n* = 3). Approximately 30 mL of methanol was added, and the sample was ultrasonicated for 10 min, followed by standing at room temperature to allow cooling. The volume was then adjusted to 50 mL with methanol, and the resulting solution was diluted and analyzed using the same procedure described for the content uniformity testing. The percent assays were subsequently reported.

#### 2.2.4. Dissolution Testing

The dissolution testing for the optimal CBD ODTs (*n* = 6) was performed using a paddle apparatus (dissolution apparatus 2). The dissolution medium consisted of 900 mL of a 1% SLS aqueous solution, which ensured sink conditions in the present study. The medium was maintained at 37°C with a stirring speed of 50 rpm. Samples were taken at specific time intervals: 5, 10, 15, 30, 45, 60, 90, 120, and 180 min. The dissolution study was extended to 180 min to adequately characterize the dissolution behavior of CBD, which is a poorly water‐soluble and highly lipophilic compound. To ensure the volume of the dissolution medium remained constant, fresh medium at 37°C was added each time a sample was collected. The obtained solutions were filtered using a 0.45‐μm nylon syringe filter before undergoing analysis via HPLC, resulting in the dissolution profile of CBD from the ODT being established.

### 2.3. HPLC Analysis of CBD

The HPLC analysis was adapted slightly from prior research [23, 24] and conducted using an Agilent 1260 Infinity II system (Agilent Technologies, Inc., CA, USA), which included an autosampler and a photodiode array detector. The separation process utilized a Poroshell 120 EC‐C18 analytical column (4.6 × 150 mm, 4 μm) that was maintained at a temperature of 25°C. The mobile phase for the analysis contained a mixture of water and methanol in a 17:83 v/v ratio, and the flow rate was set to 1 mL/min using an isocratic method. The injection volume was 10 μL, and the photodiode array detector monitored the signal at a wavelength of 222 nm. The HPLC method was validated according to the ICH Q2(R2) guidelines. Briefly, seven concentration levels of CBD standard (1.5625–100 μg/mL) were prepared with methanol as the solvent. Validation parameters included linear response, evaluated through the linear equation, test range, and coefficient of determination (*R* ^2^); specificity; lower limit ranges assessed by determining the detection limit (DL) and quantitation limit (QL) by signal‐to‐noise ratio, along with precision and accuracy (Supporting information, Figure S1 and Tables S1‐S2).

## 3. Results

### 3.1. QTPP

The identified QTPPs directly influence the quality of CBD ODTs, with their respective targets and justifications summarized in Table 3.

**TABLE 3:** QTPP for CBD ODTs.

QTPP elements		Target	Justification
Dosage form/dosage design		ODTs	To develop a new dosage form of CBD

Route of administration		Oral	Consistent with commercial products such as Epidiolex (CBD oral solution) and Xativa (CBD wafer tablet).

Dosage strength		10 mg of CBD	In accordance with the literature [19, 20], this dosage strength is classified as a low dose of CBD, which has been reported to be effective in the treatment of anxiety [25–30]. The selected dose in the present study was primarily intended for early‐stage formulation development and proof‐of‐concept evaluation. The higher doses of CBD may be required for adult patients, depending on the clinical indication, whereas the selected low dose may also apply to pediatric applications.

Pharmacokinetics		• *C*_max_: 389.17 ± 153.23 ng/mL	Bioequivalence to the commercial product. Refer to the publications [31–33].
		• AUC: 1542.19 ± 488.04 ng/mL•h	
		• Elimination half‐life: 2–5 days (repeated oral administration)	
		• Bioavailability (oral): ∼6%	
		• *T*_max_: 0–4 h	
		• Increased Cmax with food and lipid‐based formulations	
		• 7‐COOH‐CBD is a major metabolite	
		• 7‐OH‐CBD is an active metabolite	

Stability		A minimum shelf‐life of 24 months at room temperature.	Equivalent to or superior to commercial products.

Product quality attributes	Physical attributes	White color, circular, flat beveled edge, plain on both sides	
	Identification	Meet the requirements of European Pharmacopoeia (EP) 11.5 (infrared absorption spectrophotometry and specific optical rotation) and USP (HPLC) [34]	
	Assay	CBD content is 90%–110% of the stated amount.	
	Uniformity of dosage unit	AV of content uniformity not exceeding 15.0: USP <905> uniformity of dosage units [35]	
	Disintegration	Not more than 30 s according to US FDA [36].	
	Dissolution	Currently, there is no established Q value for the dissolution testing of CBD ODTs. However, 1% SLS solution was employed as the dissolution medium to enable discrimination of drug dissolution, given that CBD is a poorly water‐soluble compound [37].	
	Degradation products	CBD is thermolabile and highly susceptible to oxidation, photodegradation, and alkaline hydrolysis [23, 38, 39].	
	Microbial limits	Meet the requirements of USP: <61> Microbiological Examination of Nonsterile Products: Microbial Enumeration Tests [35]	
	Heavy metals	Meet the requirements of USP: <232> Elemental Impurities‐Limits [35]	
	Residual solvents	Meet the requirements of USP: <467> Residual Solvents [35]	

Container closure system		Stored in an airtight container such as an aluminum/aluminum blister. Pack sizes contained 10 tablets.	The ODT formulation contained MCC, a hygroscopic excipient [40]; therefore, the packaging should protect the product from environmental moisture.

The QTPP elements were defined to ensure that the developed CBD ODT meets the intended clinical and patient‐centered objectives. The dosage strength was established to provide consistent therapeutic exposure, while the dosage form selection as an ODT was justified to enhance patient compliance and facilitate administration without water. Targets for mechanical integrity were specified to ensure adequate handling and packaging robustness. Rapid disintegration was defined as a key performance criterion to support the prompt onset of action and acceptable in‐mouth behavior. In addition, assay and content uniformity targets were aligned with pharmacopeial standards to guarantee dose accuracy and product reliability. Collectively, these QTPP elements guided subsequent identification of CQAs and formulation optimization.

### 3.2. CQAs

The CQAs derived from the QTPP directly impact the safety and efficacy of CBD ODTs, with their respective targets and justifications summarized in Table 4.

**TABLE 4:** CQAs for CBD ODTs.

Quality attributes of the product		Target	Is this a CQA?	Justification
Physical attributes	Appearance	White color, circular, flat beveled edge, plain on both sides	No	Appearance, odor, size, and score configuration do not affect safety and efficacy, but size can influence patient acceptability.
	Odor	There is no odor in this formulation	No	
	Size	Diameter approximately 9.7 mm	No	
	Score configuration	Unscored	No	
	Friability	Not more than 1.0%	No	The friability is controlled at a maximum of 1.0%, ensuring that it does not influence the assay results.
	Hardness	4–6 kgf	Yes	Tablet hardness influences the disintegration process of the tablets, which in turn impacts their dissolution. Consequently, this can affect the overall efficacy of the medication.

Identification		Conform to EP or USP	Yes	Identification is essential for ensuring both the safety and efficacy of the product. This CQA will be assessed upon receipt of the raw materials to ensure that the appropriate pharmaceutical substance is used throughout the manufacturing process.

Assay		90%–110% of CBD	Yes	The assay is critical for ensuring both the safety and efficacy of the product. However, given that this formulation represents a low‐dose strength, the assay outcome is more likely to impact efficacy rather than safety.

Uniformity of dosage unit (content uniformity)		Conform to USP <905> Uniformity of Dosage Units	Yes	Content uniformity is a critical quality attribute to ensure both safety and efficacy. However, in the case of this low‐dose strength, variability in content is more likely to affect efficacy than safety.

Disintegration		Not more than 30 s (conform to US FDA definition)	Yes	Disintegration influences dissolution, which in turn affects bioavailability, potentially impacting both safety and efficacy. Nevertheless, for this low‐dose formulation, variations in content are more likely to impact efficacy rather than safety.

Dissolution		No standard criteria value for this product	Yes	Dissolution influences bioavailability, which can subsequently affect both safety and efficacy. Nevertheless, for this low‐dose formulation, variations in content are more likely to impact efficacy rather than safety.

Degradation product		Limit of CBD impurity J conforms to EP	Yes	The presence of degradation products and impurities may compromise the safety and efficacy of the formulation.

Microbial limits		Conform to USP <61> Microbiological Examination of Nonsterile Products: Microbial Enumeration Tests	Yes	Microbial contamination may lead to safety concerns in the final product.

Heavy metals		Conform to <232> Elemental Impurities‐Limits	Yes	Heavy metals may lead to safety concerns in the final product.

Residual solvents		Conform to USP <467> Residual Solvents	Yes	Residual solvents may lead to safety concerns in the final product.

Tablet hardness was defined to ensure sufficient mechanical strength during manufacturing, packaging, and handling. DT was established as a critical functional attribute to guarantee rapid tablet disintegration in the oral cavity, which is essential for patient acceptability and onset of action. Friability was included to assess resistance to abrasion and mechanical stress. Assay and content uniformity were specified to ensure dose accuracy and therapeutic consistency. Together, these CQAs represent key attributes governing both structural integrity and in‐use performance of the final product.

### 3.3. Initial Risk Assessment

An initial risk assessment was conducted for both the CBD raw material and the formulation and process parameters. The risk levels of various attributes in relation to each CQA, along with their justifications, are presented in Tables 5, 6, 7, 8.

**TABLE 5:** Initial risk assessment of CBD raw material attributes.

Product CQAs	CBD raw material attributes						
	Particle size distribution	Flow properties	Hygroscopicity	Moisture content	Solubility	Residual solvents	Chemical stability
Assay	High	High	Low	Low	Low	Low	High
Uniformity of dosage units (content uniformity)	High	High	Low	Low	Low	Low	High
Hardness	Low	Low	Low	Low	Low	Low	Low
Disintegration	Low	Low	Low	Low	Low	Low	Low
Dissolution	High	Low	Low	Low	Medium	Low	Medium
Degradation products	Low	Low	Low	Low	Low	Low	High

**TABLE 6:** Justification for the initial risk assessment of the CBD raw material attributes.

Variables	Product CQAs	Justification
Particle size distribution	Assay	Particle size distribution influences the homogeneity of the powder mixture, particularly because CBD is used at a low dose. Variability in particle size may lead to content nonuniformity and impact the assay. The risk is high.
	Content uniformity	
	Hardness	Particle size distribution does not impact hardness and disintegration. The risk is low.
	Disintegration	
	Dissolution	Particle size distribution affects the homogeneity of drug dissolution, as variations in particle size can influence the surface area available for dissolution and the rate at which the drug dissolves. The risk is high.
	Degradation products	Particle size distribution does not impact degradation product formation. The risk is low.

Flow properties	Assay	The flow properties of CBD directly impact assay and content uniformity, as CBD exhibits poor flowability and is used at a low dose. Inadequate flow characteristics can lead to drug content inconsistency during the manufacturing process. The risk is high.
	Content uniformity	
	Hardness	Flow properties do not significantly impact tablet hardness, disintegration, dissolution, or degradation product formation. The risk is low.
	Disintegration	
	Dissolution	
	Degradation products	

Hygroscopicity	Assay	CBD is a nonhygroscopic substance. Consequently, it does not significantly impact critical quality attributes such as assay, content uniformity, hardness, disintegration, dissolution, or degradation product formation. The risk is low.
	Content uniformity	
	Hardness	
	Disintegration	
	Dissolution	
	Degradation products	

Moisture content	Assay	The moisture content of CBD was controlled within specified limits. Consequently, it does not exert a significant impact on critical quality attributes such as assay, content uniformity, hardness, disintegration, dissolution, or degradation product formation. The risk is low.
	Content uniformity	
	Hardness	
	Disintegration	
	Dissolution	
	Degradation products	

Solubility	Assay	Solubility does not impact assay and content uniformity, as the selected solvent ensures the complete dissolution of CBD. The risk is low.
	Content uniformity	
	Hardness	Solubility does not impact hardness and disintegration. The risk is low.
	Disintegration	
	Dissolution	Solubility directly affects dissolution; however, the selection of an appropriate dissolution medium is essential to ensure sink conditions during testing. The risk is medium.
	Degradation products	Solubility does not impact degradation product formation. The risk is low.

Residual solvents	Assay	Residual solvents are typically controlled within stringent limits and are present at low levels. Consequently, they do not exert a significant impact on critical quality attributes such as assay, content uniformity, hardness, disintegration, dissolution, or degradation product formation. The risk is low.
	Content uniformity	
	Hardness	
	Disintegration	
	Dissolution	
	Degradation products	

Chemical stability	Assay	Chemical stability directly influences the amount of CBD present in the formulation; therefore, it impacts both assay and content uniformity. The risk is high.
	Content uniformity	
	Hardness	Chemical stability does not impact hardness and disintegration. The risk is low.
	Disintegration	
	Dissolution	Chemical stability influences the amount of CBD present in the dissolution medium. The risk is medium.
	Degradation products	Chemical stability is directly related to degradation product formation. The risk is high.

**TABLE 7:** Initial risk assessment of formulation and process parameters.

Product CQAs	Formulation variables						Process variable
	Spray‐dried mannitol	MCC	CCS	Fumed silica	Sucralose	MgSt	Compression force
Assay	Low	Medium	Low	Low	Low	Low	Low
Uniformity of dosage units (content uniformity)	Low	High	Low	Low	Low	Low	Low
Hardness	High	High	Low	Low	Low	Low	High
Disintegration	Low	High	High	Low	Low	Low	High
Dissolution	Low	High	High	Low	Low	Low	High
Degradation products	Low	Low	Low	Low	Low	Low	Low

Abbreviations: CCS = croscarmellose sodium, CQAs = critical quality attributes, MCC = microcrystalline cellulose, MgSt = magnesium stearate.

**TABLE 8:** Justification for the initial risk assessment of the formulation and process parameters of CBD ODTs.

Variables	Product CQAs	Justification
Spray‐dried mannitol	Assay	Spray‐dried mannitol does not affect the assay and content uniformity. The risk is low.
	Content uniformity	
	Hardness	Mannitol alone does not provide sufficient tablet hardness for ODTs. The risk is high.
	Disintegration	Spray‐dried mannitol exhibits rapid disintegration and dissolution upon contact with water. The risk is low.
	Dissolution	
	Degradation products	There are no documented instances, suggesting that mannitol facilitates the degradation of CBD. Additionally, mannitol is utilized as an ingredient in the commercial formulation of CBD wafer tablets. The risk is low.

MCC	Assay	MCC exhibits poor flowability, which may negatively impact the assay as a result of inconsistent powder flow. The risk is medium.
	Content uniformity	MCC exhibits poor flowability, which may adversely affect content uniformity due to inconsistent powder flow. Since content uniformity is assessed on individual tablets, MCC has a greater impact on content uniformity than on assay results. The risk is high.
	Hardness	MCC acts as a binder that can increase tablet hardness, which is also associated with disintegration and dissolution behavior. The risk is high.
	Disintegration	
	Dissolution	
	Degradation products	There are no documented instances, suggesting that MCC facilitates the degradation of CBD. Additionally, MCC is utilized as an ingredient in the commercial formulation of CBD wafer tablets. The risk is low.

CCS	Assay	As CCS is used in a low quantity, it does not impact assay, content uniformity, or hardness. The risk is low.
	Content uniformity	
	Hardness	
	Disintegration	CCS is a superdisintegrant that directly influences tablet disintegration, which consequently affects dissolution. The risk is high.
	Dissolution	
	Degradation products	There are no documented instances, suggesting that CCS facilitates the degradation of CBD. Additionally, CCS is utilized as an ingredient in the commercial formulation of CBD wafer tablets. The risk is low.

Fumed silica	Assay	Fumed silica is used as a glidant but in a small quantity; therefore, it does not impact assay, content uniformity, hardness, disintegration, or dissolution. The risk is low.
	Content uniformity	
	Hardness	
	Disintegration	
	Dissolution	
	Degradation products	There are no documented instances, suggesting that fumed silica facilitates the degradation of CBD. Additionally, fumed silica is utilized as an ingredient in the commercial formulation of CBD wafer tablets. The risk is low.

Sucralose	Assay	Sucralose is used in a small quantity and does not impact assay, content uniformity, hardness, disintegration, or dissolution. The risk is low.
	Content uniformity	
	Hardness	
	Disintegration	
	Dissolution	
	Degradation products	There are no documented instances, suggesting that sucralose facilitates the degradation of CBD. Additionally, sucralose is utilized as an ingredient in the commercial formulation of CBD wafer tablets. The risk is low.

MgSt	Assay	MgSt is used in a small quantity. It does not impact assay, content uniformity, hardness, disintegration, or dissolution. The risk is low.
	Content uniformity	
	Hardness	
	Disintegration	
	Dissolution	
	Degradation products	There are no documented instances, suggesting that MgSt facilitates the degradation of CBD. Additionally, MgSt is utilized as an ingredient in the commercial formulation of CBD wafer tablets. The risk is low.

Compression force	Assay	Compression force does not impact assay or content uniformity. The risk is low.
	Content uniformity	
	Hardness	Compression force directly affects tablet hardness, which is consequently associated with tablet disintegration and dissolution behavior. The risk is high.
	Disintegration	
	Dissolution	
	Degradation products	Compression force is not related to the formation of degradation products. The risk is low.

Abbreviations: CCS = croscarmellose sodium, CQAs = critical quality attributes, MCC = microcrystalline cellulose, MgSt = magnesium stearate.

The risk ranking presented in Tables 5, 6, 7, 8 was established based on a qualitative assessment of the potential impact of material attributes and formulation and process parameters on the identified CQAs. Factors considered to have a direct and significant influence on mechanical integrity and disintegration performance were assigned higher risk levels. Parameters categorized as high risk were subsequently identified as critical and selected for further evaluation in the DoE study. This structured initial risk assessment ensured that the experimental investigation focused on scientifically justified and potentially impactful variables.

The authors acknowledge that microbial limits, heavy metal limits, and residual solvents are considered to be CQAs for the drug product. However, these attributes were not evaluated at this stage, as the study is currently in the research and development (R&D) phase. Therefore, the CQAs included in the initial risk assessment were limited to assay, content uniformity, hardness, disintegration, dissolution, and degradation products.

### 3.4. Optimal Formulation

Table 9 summarizes the experimental design, including the studied factors and their investigated ranges. The independent variables were compression force (*X*_1_), MCC content (*X*_2_), and CCS content (*X*_3_). The selected ranges were defined based on preliminary formulation trials and the outcomes of the initial risk assessment to ensure practical manufacturability while maintaining potential impact on the identified CQAs. The responses evaluated in this study were tablet hardness (*Y*_1_), DT (*Y*_2_), and friability (*Y*_3_), representing key mechanical and functional attributes of the CBD ODT. These responses were chosen to reflect both structural robustness and in‐mouth performance, thereby supporting balanced formulation optimization.

**TABLE 9:** Responses for each experimental run of the Box–Behnken design.

Standard order	Run order	Factors			Responses		
		Force (psi)	MCC (%)	CCS (%)	Hardness (kgf, *n* = 10)	DT (s, *n* = 6)	Friability (%, 13 tablets)
17	1	1500	40	3	4.97 ± 0.32	12.16 ± 0.23	0.31
11	2	1500	30	5	4.96 ± 1.04	16.43 ± 0.95	0.19
9	3	1500	30	1	5.42 ± 0.52	14.62 ± 1.31	0.41
2	4	1750	30	3	6.90 ± 0.45	18.13 ± 0.95	0.42
14	5	1500	40	3	6.02 ± 0.71	15.22 ± 0.36	0.30
1	6	1250	30	3	4.43 ± 0.29	17.42 ± 0.64	0.37
6	7	1750	40	1	6.56 ± 0.81	18.60 ± 1.24	0.29
13	8	1500	40	3	6.13 ± 0.45	15.90 ± 0.71	0.20
4	9	1750	50	3	7.26 ± 0.84	18.87 ± 1.19	0.19
7	10	1250	40	5	5.16 ± 0.42	19.06 ± 0.53	0.29
8	11	1750	40	5	8.03 ± 0.66	24.12 ± 0.68	0.13
10	12	1500	50	1	6.35 ± 0.97	21.33 ± 0.83	0.18
15	13	1500	40	3	6.12 ± 0.80	17.54 ± 0.97	0.20
3	14	1250	50	3	5.31 ± 0.61	17.52 ± 0.53	0.14
5	15	1250	40	1	5.83 ± 0.73	17.20 ± 0.60	0.13
16	16	1500	40	3	5.97 ± 0.58	17.27 ± 0.64	0.25
12	17	1500	50	5	7.26 ± 0.59	22.21 ± 0.91	0.15

The coded equations were derived using normalized factor levels (−1 to +1), allowing direct comparison of coefficient magnitudes, where larger coefficients indicate greater influence on the response. The actual equations were generated using real unit values for prediction under practical operating conditions. As shown in Table 10, compression force exerted the strongest effect on tablet hardness, whereas MCC was the dominant factor influencing both DT and friability. CCS contributed notably to disintegration behavior, particularly through its quadratic effect, indicating a nonlinear influence at higher levels. Interaction terms further demonstrated that combined factor effects were relevant to friability.

**TABLE 10:** Equations in coded and actual values for each response derived from the Box–Behnken design.

Responses	Coded equations	Actual equations
Hardness	*Y*_1_ = 6.04 + 1.00*X*_1_ + 0.56*X*_2_ + 0.16*X*_3_	*Y*_1_ = −2.44 + 0.004*X*_1_ + 0.06 + 0.08*X*_1_ *X*_2_
DT	$Y_{2}=15.621.071.671.260.160.920.231.730.632.39+X_{1}+X_{2}+X_{3}+X_{1}X_{2}+X_{1}X_{3}-X_{2}X_{3}+{X_{1}}^{2}+{X_{2}}^{2}+{X_{3}}^{2}$	$Y_{2}=89.260.090.405.240.000060.0020.010.000030.0060.60-X_{1}-X_{2}-X_{3}+X_{1}X_{2}+X_{3}-X_{2}X_{3}+{X_{1}}^{2}+{X_{2}}^{2}+{X_{3}}^{2}$
Friability	*Y*_3_ = 0.24 + 0.01*X*_1_ − 0.09*X*_2_ − 0.03*X*_3_ − (5.05 × 10^−17^)*X*_1_ *X*_2_ − 0.08*X*_1_ *X*_3_ + 0.05*X*_2_ *X*_3_	*Y*_3_ = 0.15 + 0.0005*X*_1_ − 0.02*X*_2_ + 0.13*X*_3_ + (9.67 × 10^−21^)*X*_1_ *X*_2_ − 0.0002*X*_1_ *X*_3_ + 0.002*X*_2_ *X*_3_

*Note:* *Y*_1_, *Y*_2_, and *Y*_3_ are the hardness, DT, and friability, respectively. *X*_1_, *X*_2_, and *X*_3_ are compression force, quantity of MCC, and quantity of CCS, respectively.

**FIGURE 1:**
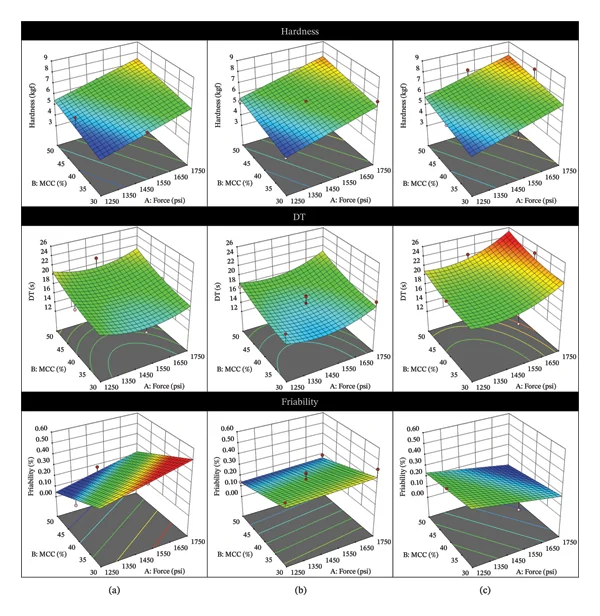
Response surface plots of hardness, DT, and friability at CCS levels of (a) 1%, (b) 3%, and (c) 5%.

Response surface plots of hardness, DT, and friability are shown in Figure 1. Increasing compression force increased tablet hardness and prolonged DT, reflecting enhanced compaction and reduced porosity. For friability, compression force increased friability at low CCS levels but decreased friability at high CCS levels, indicating an interaction effect between compression force and CCS. Increasing MCC increased hardness, slightly prolonged DT, and decreased friability due to improved interparticulate bonding. Increasing CCS slightly increased hardness, prolonged DT, and decreased friability, with curvature observed in the response surface confirming its nonlinear influence on disintegration behavior at higher levels.

Based on ANOVA data in Table 11, compression force and MCC content were significant terms for hardness, confirming that increased compaction pressure and binder level enhance interparticulate bonding, reduce porosity, and consequently improve mechanical strength. No significant linear terms were identified for DT, suggesting that DT is governed by combined or nonlinear effects of formulation factors rather than a single dominant variable. In addition, the ANOVA model for DT was not statistically significant under the investigated experimental conditions. Therefore, the interpretation of the DT response and its predictive capability should be approached cautiously compared with the hardness and friability models. For friability, MCC content showed a significant negative effect, indicating improved resistance to abrasion with higher binder levels due to strengthened tablet structure. In addition, the significant interaction between compression force and CCS highlights that mechanical robustness depends on the balance between compaction intensity and superdisintegrant level. The lack of fit was insignificant for all responses, confirming model adequacy and predictive reliability.

**TABLE 11:** ANOVA data for each response of the Box–Behnken design.

Source	Sum of squares	df	Mean square	*F*‐value	*p*‐value
*Hardness*					
Model	10.7300	3	3.5800	11.7200	0.0005^∗^
A‐Force	8.0400	1	8.0400	26.3300	0.0002^∗^
B‐MCC	2.5000	1	2.5000	8.1800	0.0134^∗^
C‐CCS	0.1953	1	0.1953	0.6397	0.4382
Residual	3.9700	13	0.3053		
Lack of fit	3.0000	9	0.3334	1.3800	0.4043
Pure error	0.9687	4	0.2422		
Cor total	14.7000	16			

*DT*					
Model	89.5200	9	9.9500	1.7100	0.2463
A‐Force	9.0700	1	9.0700	1.5600	0.2520
B‐MCC	22.2100	1	22.2100	3.8200	0.0917
C‐CCS	12.6800	1	12.6800	2.1800	0.1836
AB	0.1024	1	0.1024	0.0176	0.8982
AC	3.3500	1	3.3500	0.5752	0.4730
BC	0.2162	1	0.2162	0.0371	0.8527
*A* ^2^	12.6300	1	12.6300	2.1700	0.1842
*B* ^2^	1.7000	1	1.7000	0.2914	0.6061
*C* ^2^	24.1500	1	24.1500	4.1500	0.0811
Residual	40.7500	7	5.8200		
Lack of fit	22.1300	3	7.3800	1.5900	0.3254
Pure error	18.6200	4	4.6500		
Cor total	130.2700	16			

*Friability*					
Model	0.1103	6	0.0184	5.2100	0.0112^∗^
A‐Force	0.0013	1	0.0013	0.3540	0.5651
B‐MCC	0.0666	1	0.0666	18.8600	0.0015^∗^
C‐CCS	0.0078	1	0.0078	2.2100	0.1677
AB	1.3880 × 10^−17^	1	1.3880 × 10^−17^	3.9300 × 10^−15^	1.0000
AC	0.0256	1	0.0256	7.2500	0.0226^∗^
BC	0.0090	1	0.0090	2.5600	0.1410
Residual	0.0353	10	0.0035		
Lack of fit	0.0242	6	0.0040	1.4600	0.3727
Pure error	0.0111	4	0.0028		
Cor total	0.1456	16			

^∗^indicates a significant value.

**FIGURE 2:**
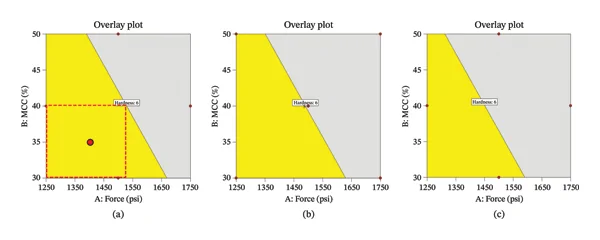
Design spaces where hardness was 4–6 kgf, DT ≤ 30 s, and friability ≤ 1% at CCS levels of (a) 1%, (b) 3%, and (c) 5%. The red dashed‐line square indicates the control space, and a red circular marker within the control space represents the selected optimal formulation.

Design spaces where hardness ranged from 4–6 kgf, DT ≤ 30 s, and friability ≤ 1% are illustrated in Figure 2. Figure 2a–c represent the graphical overlay plots for each response criterion and collectively define the multidimensional region where all responses simultaneously meet the predefined specifications. The similarities observed across different CCS levels indicate that acceptable performance can be achieved within comparable operating ranges; therefore, the design space at a low quantity of CCS was selected to maintain formulation simplicity and efficiency. From this selected design space, a control space (normal operating range) was established to ensure consistent product quality within defined boundaries. Formulations within this space were considered optimal. In this case, the optimal formulation consisted of 35% MCC and 1% CCS, with a compression force of 1400 psi, representing a balanced condition between mechanical robustness and rapid disintegration.

Three lots of the optimal formulation were prepared to verify the accuracy of the model predictions. The results showed that all actual values closely matched the predicted values and fell within the 95% CI (Table 12), indicating that the model predictions were accurate and reliable.

**TABLE 12:** Verification of the predictive model: comparison of predicted and experimental values with 95% CI.

Responses	Predicted values	Experimental values			95% CI (lower–upper)
		Lot 1	Lot 2	Lot 3	
Hardness (kgf, *n* = 10)	5.20	4.97	4.68	4.69	4.62–5.78
DT (s, *n* = 6)	16.21	15.15	13.37	15.16	12.37–20.06
Friability (%, 13 tablets)	0.31	0.25	0.26	0.34	0.23–0.39

Each lot of the optimal formulation was sampled to determine assay and content uniformity in accordance with the USP procedure. The results, Table 13, showed that assay values were within 90%–110% of the label claim, and the content uniformity exhibited an AV below the maximum allowable limit (L1), indicating that both assay and content uniformity were acceptable at this stage.

**TABLE 13:** Assay and content uniformity of the optimal CBD ODTs.

Parameters		Lot		
		1	2	3
Assay (%, mean ± SD)		92.72 ± 1.73	94.61 ± 1.29	91.88 ± 0.85

Content uniformity (%, *n* = 10)	*X̅*	92.45	97.84	93.38
	*s*	3.46	5.04	3.63
	AV = |*M* − *X̅*| + ks	14.34	12.76	13.82
	L1	15.0		
	Summary	AV < L1		

*Note: X̅* is the mean of individual content values, expressed as a percentage of the label claim; s or SD is the standard deviation of the sample; AV is the acceptance value; *M* is the reference value, where *M* = 98.5% when *X̅* < 98.5%; *k* is the acceptability constant, with *k* = 2.4 for *n* = 10; and L1 is the maximum allowed acceptance value, set at L1 = 15.0.

**FIGURE 3:**
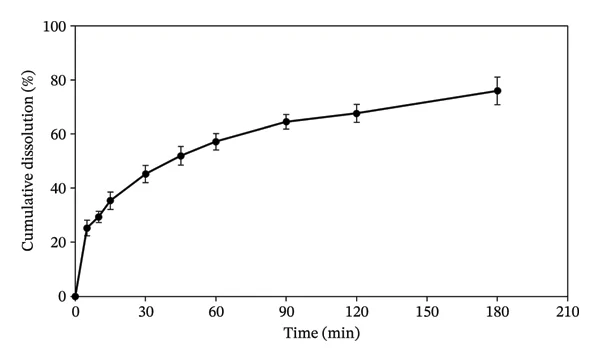
Dissolution profile of CBD from CBD ODTs in 1% SLS solution (*n* = 6).

Lot 1 of the optimal formulation was sampled to evaluate dissolution in 1% SLS aqueous solution. The dissolution profile of CBD from the CBD ODTs is shown in Figure 3. CBD was gradually released into the medium, with 75.98% dissolved within 3 h of testing.

## 4. Discussion

ODTs are designed to disintegrate rapidly in the oral cavity without the need for water, thereby improving patient convenience and compliance, particularly in populations with swallowing difficulties. The US Food and Drug Administration generally recommends a DT of not more than 30 s for ODT classification [36]. In addition to rapid disintegration, ODTs must possess adequate mechanical strength to withstand handling, packaging, and transportation. Excessive hardness may delay disintegration, whereas insufficient mechanical integrity can result in high friability and tablet breakage. Therefore, an optimal balance between mechanical robustness and rapid in‐mouth disintegration is essential. These performance requirements are widely reported in the literature as critical determinants of ODT quality, patient acceptability, and therapeutic reliability. In this context, the selected responses—hardness, DT, and friability—represent key quality indicators for ensuring both structural integrity and functional performance of CBD ODTs. Nevertheless, the authors acknowledge that rapid tablet disintegration does not necessarily correspond to rapid systemic absorption, particularly for highly lipophilic compounds such as CBD. Therefore, additional studies involving permeability, pharmacokinetic, or in vivo evaluation would be necessary to further establish the translational performance of the developed CBD ODT formulation.

QbD offers a systematic framework for optimizing the formulation development of CBD ODTs, primarily through QTPP definition and CQAs identification. Methodologies such as the Box–Behnken design and risk management strategies can help address challenges associated with CBD delivery. In addition, predictive models may facilitate formulation optimization and support predefined quality targets [41]. In the present study, the QbD approach enabled the identification of compression force, MCC, and CCS as critical variables influencing mechanical strength and tablet performance, leading to the establishment of a statistically justified design space and control space. Previous CBD ODT studies mainly emphasized formulation optimization or dissolution enhancement. In contrast, the present work incorporated broader QbD elements, including systematic risk assessment and definition of operating ranges associated with predefined quality targets. This strategy provided additional process understanding and supported more structured formulation development. This reduces formulation uncertainty and minimizes reliance on empirical adjustments. The developed regression models provide quantitative guidance for selecting operating conditions within the investigated experimental ranges, which may support future formulation optimization studies. The structured QbD framework may support future CBD ODT formulation development. However, additional studies related to process scale‐up, manufacturability, and *in vivo* performance would still be required. Furthermore, manufacturing robustness studies, process validation, and accelerated stability investigations were beyond the scope of the present laboratory‐scale formulation development study. Nevertheless, the established design space and control space may provide useful guidance for subsequent process development and future scale‐up investigations.

The investigated variables were selected based on their mechanistic roles in tablet compaction and disintegration. Compression force was included because of its direct impact on interparticulate bonding, porosity, and mechanical integrity. MCC was selected because its plastic deformation behavior and binder functionality influence hardness and friability. CCS was incorporated as a superdisintegrant responsible for water uptake and tablet breakdown. Hardness, DT, and friability were selected as representative critical attributes of CBD ODTs, reflecting mechanical robustness and in‐mouth performance. Together, these responses provide a balanced evaluation of structural strength and functional behavior.

The dose of 10 mg CBD incorporated into each tablet was followed by the previous study related to CBD ODTs [19, 20]. This dosage strength is classified as a low dose of CBD, which has been reported to be effective in the treatment of anxiety [25–29]. In addition, the selection of a low‐dose formulation was considered appropriate for the present proof‐of‐concept QbD study. Low‐dose tablets are generally more sensitive to formulation and process variability, particularly with respect to content uniformity and blend homogeneity. Therefore, the selected dose provided a suitable model for early‐stage pharmaceutical development. It also allowed evaluation of formulation robustness and process understanding. The authors acknowledge that this dose may be relatively low for adult patients in certain therapeutic settings. Multiple dosage units may be required to achieve the desired therapeutic effect. In contrast, the selected dose may also apply to pediatric applications. In addition, the other indications required higher doses of CBD [42], and several clinical studies investigating anxiety‐related disorders have commonly employed higher oral doses of CBD. Therefore, the present formulation should primarily be considered a proof‐of‐concept and early‐stage pharmaceutical development model rather than a finalized clinical dosing strategy for adult patients. Further studies may be required to evaluate the applicability of the present formulation strategy to higher dose CBD products.

The authors acknowledge certain limitations in this study, particularly the restricted availability of CBD due to its high cost. For instance, standard friability testing typically requires approximately 6.5 g of tablets (equivalent to 26 tablets). However, this requirement could not be fully achieved in the present study because the research was conducted at the R&D stage; the number of tablets was reduced to 13 to allow for comparative evaluation of different formulations while minimizing material usage. The authors acknowledge that this deviation from pharmacopeial recommendations may reduce the robustness of the friability evaluation and mechanical characterization of the developed CBD ODTs. Moreover, tablet compression was performed using an in‐house assembled hydraulic press at the laboratory scale. Although this setup was considered suitable for preliminary formulation screening and QbD‐based optimization, differences in compression dynamics and process consistency may exist compared with industrial rotary tablet presses. Therefore, further studies using commercial‐scale manufacturing equipment would be necessary to confirm reproducibility, compression consistency, and scalability of the developed CBD ODT formulation. In addition, stability evaluation was not included in the present study despite the known susceptibility of CBD to environmental stress conditions, including oxidation, heat, and light exposure. Therefore, further studies involving accelerated and long‐term stability testing would be necessary to establish the pharmaceutical stability and shelf‐life of the developed CBD ODT formulation. In addition, palatability and taste‐masking performance were not evaluated in the present study, despite their importance for patient acceptability of ODT formulations. This consideration may be particularly relevant for cannabinoid‐containing products because of their characteristic taste properties. Therefore, future studies involving sensory evaluation and taste‐masking optimization would be beneficial.

The present study aligns with earlier findings, which indicated that increasing compression force commonly leads to decreased friability, along with heightened tablet hardness and prolonged DT [22, 43, 44]. Specifically, higher compression forces tend to result in lower friability because they promote closer bonding among powder particles, creating a denser tablet with enhanced mechanical strength. A more compact tablet structure is inherently less vulnerable to breaking or chipping, thereby resulting in reduced friability. As the compression force rises, the tablet exhibits increased strength and cohesiveness, which diminishes its likelihood of disintegration under stress. Although several experimental runs produced hardness values outside the predefined target range of 4–6 kgf, such deviations are expected during DoE‐based formulation development because tablet properties are simultaneously influenced by multiple formulation and process variables. Importantly, the established design space enabled identification of optimized operating conditions capable of meeting the predefined quality criteria for hardness, DT, and friability. However, this study noted that compression force did not impact DT. Given that the DT of CBD ODTs is exceedingly short, the variations in compression force did not influence this parameter. In addition, the present study demonstrated that systematic evaluation of compression force together with MCC and CCS enabled identification of operating conditions that balanced mechanical robustness and rapid disintegration within the defined design space. This approach provided enhanced process understanding and improved formulation predictability despite the use of conventional ODT excipients.

In addition to compression force, MCC plays a critical role as a dry binder in the formulation. MCC undergoes plastic deformation during compression. This behavior increases interparticulate contact area and promotes hydrogen bond formation, thereby enhancing tablet hardness and mechanical integrity [45]. Consequently, increasing MCC content generally improved hardness and reduced friability due to strengthened internal bonding within the tablet matrix. MCC may also facilitate water uptake through its porous structure, thereby contributing to tablet disintegration [46]. However, higher bonding density may reduce matrix porosity and slightly prolong DT. Although relatively high MCC levels were incorporated in the formulation, the optimized CBD ODTs still demonstrated rapid DT within the predefined target range. Nevertheless, patient acceptability and mouthfeel were not evaluated in the present study and should be further investigated in future sensory evaluation studies. Additionally, variations in MCC quantity also reflected changes in spray‐dried mannitol quantity, as it was used as a filler to adjust tablet weight. Nevertheless, DT remained relatively stable across the investigated formulation conditions, suggesting that the developed CBD ODT formulation maintained rapid disintegration despite variations in compression force, MCC quantity, and CCS quantity.

This study found that increasing the quantity of CCS appeared to prolong the DT, which contrasts with the typical disintegrant behavior of CCS. This observation may be associated with formulation‐specific interactions occurring under the investigated experimental conditions, including potential changes in tablet hardness at higher CCS levels. The inherently short DT of the CBD ODTs remained relatively unaffected by variations in compression force, MCC content, and CCS content. Therefore, although an increase in CCS quantity appeared to prolong DT, the effect was not statistically significant. However, the authors acknowledge that no mechanistic investigations were performed in the present study; therefore, the exact cause of the observed CCS behavior could not be conclusively established. Moreover, a significant interaction was observed between compression force and CCS quantity in relation to friability. Specifically, increasing the compression force at a low CCS level resulted in higher friability, whereas increasing the compression force at a high CCS level led to reduced friability. This interaction between compression force and superdisintegrant has also been reported previously [47].

The sink condition for the dissolution testing was verified prior to experimentation. The present study determined the solubility of CBD in 1% SLS solution at 37°C to be 323.51 ± 22.21 μg/mL. A dissolution medium volume of 900 mL was employed. This volume was approximately 29 times greater than that required to dissolve the measured amount of CBD, thereby confirming that the dissolution conditions fulfilled the sink condition. However, the solubility observed in this study was slightly lower than that previously reported (399.8 μg/mL) [48]. The authors acknowledge that the use of 1% SLS aqueous solution as the dissolution medium may not fully represent physiological conditions and could potentially overestimate dissolution performance compared with in vivo environments. Nevertheless, the selected medium was primarily intended to ensure sink conditions and enable discriminatory assessment of formulation performance during pharmaceutical development. Therefore, future studies using more biorelevant dissolution media may provide improved translational relevance.

The observed phenomenon, where CBD dissolution tests were conducted under sink conditions yet resulted in only a 76% release over 3 h, can be attributed to several factors intrinsic to the formulation and the physicochemical properties of CBD. Although sink conditions, defined as having a dissolution medium capable of dissolving at least three times the amount of the tested drug [49], were maintained, CBD is characterized by low solubility and poor permeability [50], which can significantly limit its release in practical settings. Furthermore, formulation factors such as the presence and nature of excipients, which can play crucial roles in either enhancing or inhibiting drug solubility and dissolution rates, could also hinder achieving complete release [51, 52]. Lastly, the intrinsic lipophilicity of CBD means that, even under sink conditions, a portion of the drug may remain undissolved or interact strongly with excipients, leading to a suboptimal release profile that fails to reach 100% within the specified timeframe [53]. The authors acknowledge that the observed dissolution profile was relatively slow for an immediate‐release ODT formulation, despite the rapid tablet disintegration achieved in the present study. This finding suggests that tablet disintegration alone may not be sufficient to ensure rapid dissolution of highly lipophilic compounds such as CBD. Therefore, incorporation of dedicated solubility‐enhancing approaches, such as lipid‐based systems, amorphous solid dispersions, or other enabling technologies, may be necessary in future studies to further improve the dissolution performance and translational potential of CBD ODT formulations.

To enhance the absorption of CBD, ODTs or plain tablets can incorporate solubility‐enhanced forms of CBD, such as cyclodextrin complexes [54], liquisolid systems [19, 55], mesoporous silica‐based formulations [54], self‐emulsifying drug delivery systems [56, 57], or solid dispersion [54, 58, 59]. However, factors such as production cost, manufacturing complexity, and the extent of solubility enhancement should be carefully considered during formulation development [60–62].

## 5. Conclusions

The present study demonstrates the successful development of CBD ODTs using the QbD approach, which has proven effective in enhancing both the efficiency and robustness of the formulation process. The structured application of QbD facilitated the identification and assessment of QTPP, CQAs, and risk factors, contributing to a deeper understanding of the variables influencing formulation outcomes. The use of a Box–Behnken design provided insights into the effects of compression force, MCC, and CCS on critical tablet attributes. Results indicated that formulations within the defined design and control spaces exhibited appropriate mechanical strength, rapid DT, and low friability, aligning with established quality standards for ODTs. The optimized formulation, containing suitable levels of MCC and CCS, yielded tablets with favorable properties, including sufficient hardness, rapid disintegration, and minimal friability. These findings affirm the robustness of the design and validate the predictive capability of the computational modeling. Additionally, high content uniformity and assay performance were observed, ensuring consistent dosing and therapeutic reliability. The dissolution profile demonstrated an appreciable release of CBD over time. Overall, this study demonstrates the successful implementation of a QbD‐driven strategy for laboratory‐scale formulation development and optimization of CBD ODTs. The findings contribute to pharmaceutical development efforts involving cannabinoid‐based formulations and may provide useful guidance for future studies related to scale‐up, stability evaluation, and in vivo performance.

## Author Contributions

**Chaowalit Monton:** conceptualization, methodology, formal analysis, investigation, writing–original draft, writing–review and editing, supervision, and project administration. **Poj Kulvanich:** conceptualization, formal analysis, writing–original draft, and writing–review and editing. **Apirada Sucontphunt:** formal analysis, resource, and writing–original draft. **Jirapornchai Suksaeree:** methodology, formal analysis, investigation, and writing–original draft. **Laksana Charoenchai:** formal analysis, resource, and writing–original draft. **Thanapat Songsak:** formal analysis, resource, and writing–original draft.

## Funding

No funding was received for this manuscript.

## Conflicts of Interest

The authors declare no conflicts of interest.

## Supporting Information

Additional supporting information can be found online in the Supporting Information section.

## Supporting information


**Supporting Information** HPLC Method Validation. Figure S1 HPLC chromatograms of (a) CBD standard (50 μg/mL), (b) CBD ODT (CBD at 50 μg/mL), (c) placebo, and (d) medium (1% SLS aqueous solution). Table S1 Linear response and lower range limits. Table S2 Precision and accuracy (*n* = 3).

## Data Availability

The data supporting the findings of this study are available from the authors upon reasonable request.
