# Supplementary material for: Quality by Design–Driven Formulation Development of Cannabidiol Orally Disintegrating Tablets
Source: Scientifica (Cairo). 2026 Aug 27;2026:3553253. doi: 10.1155/sci5/3553253 (PMC13519653; doi:10.1155/sci5/3553253)
Supplement: Supplementary file 1 [file SCI5-2026-3553253-s001.pdf]

Supplementary Material

Quality by Design-Driven Formulation Development of Cannabidiol Orally Disintegrating Tablets

Chaowalit Monton [1,2,3,*], Poj Kulvanich [4], Apirada Sucontphunt [1,3], Jirapornchai Suksaeree [5], Laksana Charoenchai [1], Thanapat Songsak [2,3]

[1] *Drug and Herbal Product Research and Development Center, College of Pharmacy, Rangsit University, Pathum Thani 12000, Thailand*

[2] *Department of Pharmacognosy, College of Pharmacy, Rangsit University, Pathum Thani 12000, Thailand*

[3] *Medicinal Cannabis Research Institute, College of Pharmacy, Rangsit University, Pathum Thani 12000, Thailand*

[4] *Industrial Pharmacy Program, College of Pharmacy, Rangsit University, Pathum Thani 12000, Thailand*

[5] *Department of Pharmaceutical Chemistry, College of Pharmacy, Rangsit University, Pathum Thani 12000, Thailand*

* Corresponding author: chaowalit@rsu.ac.th (C. Monton)

HPLC Method Validation

The authors note that the HPLC method in this study was validated in accordance with ICH Q2(R2), which introduces certain terminology updates compared to ICH Q2(R1). Representative HPLC chromatograms of the CBD standard and CBD in the CBD ODT formulation are shown in Figs. S1a and S1b, respectively. The retention time of CBD was observed at 5.1 min.

The HPLC method demonstrated a linear response within the tested concentration range, with low detection limit (DL) and quantitation limit (QL), as shown in Table S1. Specificity was evaluated by comparing the HPLC chromatograms of the CBD ODT with that of the placebo. As shown in Fig. S1c, the placebo chromatogram did not exhibit any peaks at the retention time corresponding to CBD in the CBD ODT, indicating that the HPLC method is specific for the analysis of CBD in the formulation. Furthermore, the authors confirmed that the dissolution medium (1% SLS solution) did not interfere with the CBD peak, as demonstrated in Fig. S1d.

The percent relative standard deviation (%RSD) for both intraday and interday precision did not exceed 4% at 80%, 100%, and 120% of the target concentrations. Furthermore, the method exhibited acceptable accuracy, with recovery values for all concentrations falling within the range of 85–110%, as presented in Table S2. These results indicate that the HPLC method is both precise and accurate.

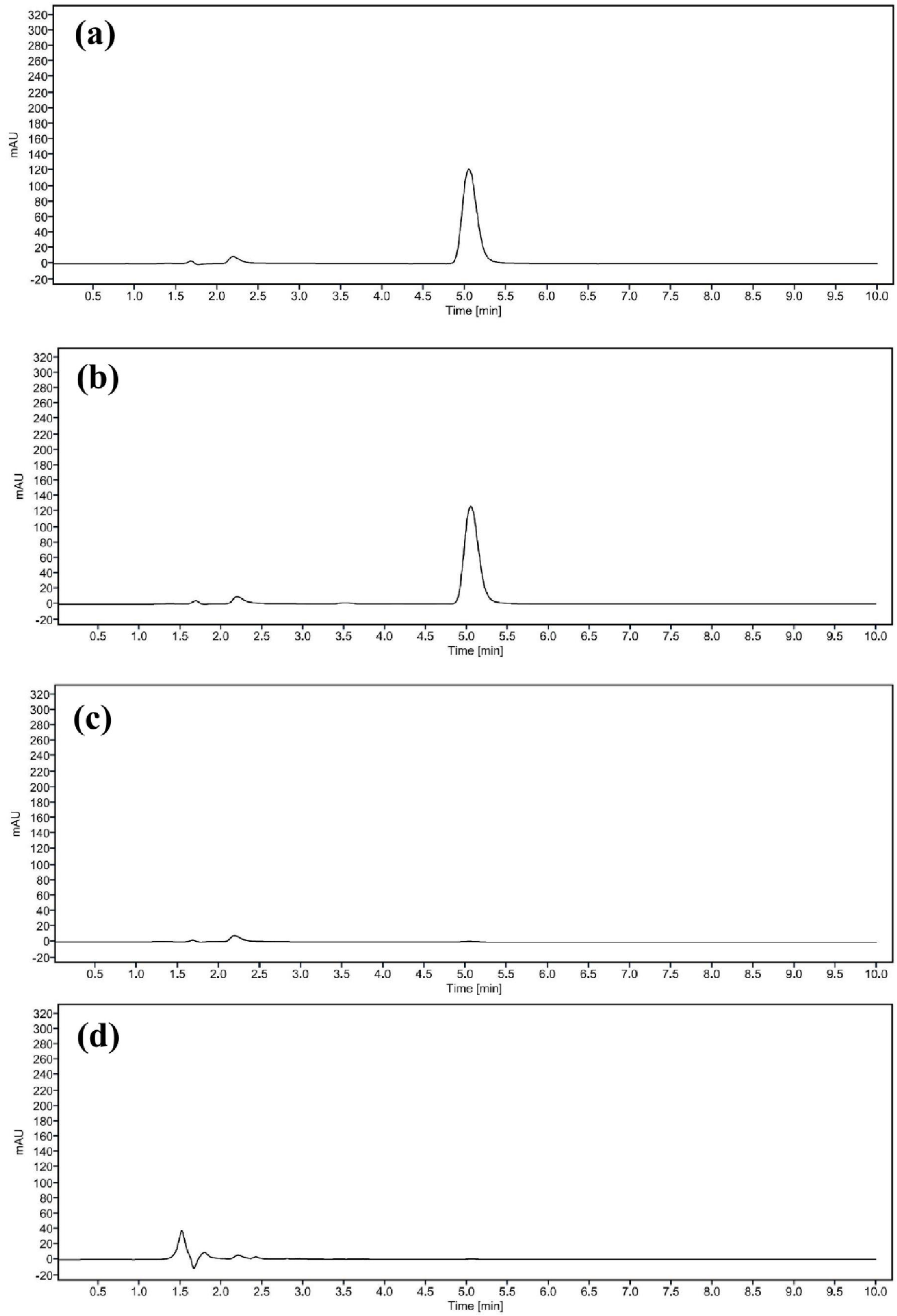

Fig. S1 HPLC chromatograms of (a) CBD standard (50 μg/mL), (b) CBD ODT (CBD at 50 μg/mL), (c) placebo, and (d) medium (1% SLS aqueous solution).

Table S1 Linear response and lower range limits.

Working range		Results
Linear response	Linear equation	y = 30.872x + 14.163
	R^2	1.0000
	Test range (µg/mL)	1.5625−100
Lower range limits	DL (µg/mL)	0.0156
	QL (µg/mL)	0.0312

Table S2 Precision and accuracy (n = 3).

Concentration (μg/mL)	Precision (RSD, %)				Spike concentration (μg/mL)	Accuracy		
	Intraday			Inter-day		Recovery (%)		
	Day 1	Day 2	Day 3			Day 1	Day 2	Day 3
40	0.20	0.27	0.27	0.23	40	87.97 ± 0.22	101.55 ± 0.34	99.83 ± 0.16
50	0.03	0.12	0.10	1.51	50	91.14 ± 0.13	101.06 ± 0.06	99.22 ± 0.10
60	0.09	0.16	0.61	0.34	60	87.40 ± 0.10	98.04 ± 0.17	97.25 ± 0.58
